# Response to comment on: Low serum testosterone is associated with an increased risk of first-time renal calculi in men without testosterone replacement therapy

**DOI:** 10.1038/s41443-025-01045-2

**Published:** 2025-04-10

**Authors:** Austin Thompson, Danly Omil-Lima, Nannan Thirumavalavan

**Affiliations:** 1 Case Western Reserve University School of Medicine, Cleveland, OH USA; 2 Fox Chase Cancer Center at Temple University, Philadelphia, PA USA; 3 University Hospitals Cleveland Medical Center, Cleveland, OH USA

**Keywords:** Risk factors, Endocrine system and metabolic diseases

We would like to thank Drs. Krishingner and Campbell for their comment regarding our manuscript assessing the risk of first-time renal calculi in hypogonadal men [1, 2]. We intended to leverage a national, multi-institutional dataset to retrospectively address and illuminate an understudied topic with uncertain results. While our study adds to the existing literature, it certainly does not bring clarity. Additional studies utilizing intentional data collection of serum hormone levels (total testosterone, free testosterone, sex hormone binding globulin (SHBG), Luteinizing hormone (LH), estrogen), testosterone-level altering therapy, and stone-related factors (serum and urine solutes, pH) are necessary to create large cohorts with robust data. Similar to the M-STONE group [3], multi-institutional collaboration would augment such data collection, allowing for multi-regional representation of hormonal and stone characteristics, which may vary in regions such as the “Stone Belt.”

Multiple factors could underlie the unclear relationship between testosterone levels and nephrolithiasis. One possible explanation requiring confirmation is a non-linear relationship between serum testosterone levels and nephrolithiasis risk. Although a physiologic explanation exists, a U-shaped relationship between serum testosterone levels and body mass index has been reported [4].

Many additional questions exist in understanding the relationship between serum hormones (extending beyond serum testosterone) and nephrolithiasis risk. Evident within the literature, diagnoses of low testosterone are increasing across men of all ages [5]. While testosterone replacement therapy (TRT) prescription trends appeared to decrease up to 2017 [5], more recent data suggest increased utilization of TRT following changes to the American Urological Association testosterone deficiency guidelines in 2018 [6]. If one humors the possibility of a non-linear association between serum testosterone levels and nephrolithiasis risk, perhaps TRT use is associated with urolithiasis risk as previously suggested [7], especially when considering compounding TRT prescriptions. If used inappropriately or without monitoring, hyperandrogenism could potentially result. We must also consider the physiologic dynamics of abrupt TRT discontinuation and the potential return to hypogonadism.

Finally, with declining serum testosterone levels in adolescents and young adult men, one must wonder if an inflection point for nephrolithiasis risk exists with respect to serum testosterone levels. Within the United States from 2008 to 2017, men aged 34–44 had the highest annual incidence of hypogonadism diagnoses [5]. Interestingly, in our study, men aged 34–44 with low testosterone had the highest risk of incident nephrolithiasis diagnosis. As men aged in our study, the risk of nephrolithiasis diagnosis in hypogonadal men declined when compared to their eugonadal counterparts [2]. Moreover, we used the AUA guideline recommendation of 300 ng/dL to define low total testosterone. Additional consideration and investigation utilizing age-specific low testosterone thresholds may provide new insights and raise additional questions [8]. As men age, the age-specific low testosterone threshold approaches the AUA recommendation of 300 ng/dL (409 ng/dL for men aged 20–24; 350 ng/dL for men aged 40–44). Of many things, the apparent question is – does the severity of hypogonadism influence the risk for nephrolithiasis? Fig. 1 suggests such a relationship but requires confirmation in studies dedicated to answering this question.

**Fig. 1:**
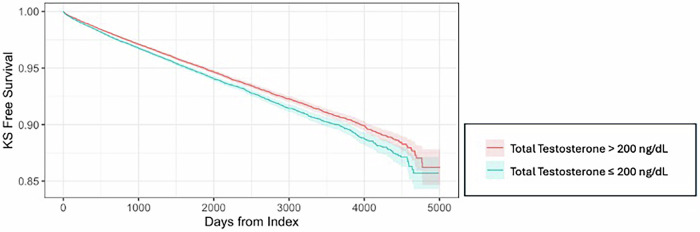
Kidney stone-free survival by total testosterone level in hypogonadal men aged ≥18. Index event is the first total testosterone level <300 ng/dL. Cohorts were split near the median total testosterone level of 210 ng/dL.

While our comment excludes a detailed physiologic discussion of the role of testosterone and additional hormones on the maintenance of male homeostasis, as highlighted by Drs. Krishingner and Campbell, Urologists, are uniquely positioned to progress our understanding of endocrinological factors that may predispose to kidney stones [1]. Multi-institutional collaboration reflective of the predominant geographic divisions of the United States may facilitate the creation of intentionally built datasets tasked to rigorously investigate the association between hormone levels and stone predisposition.

## Data Availability

The data in our comment is available upon responsible request. De-identified data from the TriNetX Research Network (Cambridge, MA, USA) was used to create Fig. 1. The MetroHealth System Institutional Review Board has deemed using TriNetX in this way exempt from IRB review.
